# How is increased selectivity of medical school admissions associated with physicians’ career choice? A Japanese experience

**DOI:** 10.1186/s12960-020-00480-0

**Published:** 2020-05-27

**Authors:** Reo Takaku

**Affiliations:** Graduate School of Economics, Hitotsubashi University, 2-1 Naka, Kunitachi, Tokyo, 186-8601 Japan

## Abstract

**Background:**

During the long-lasting economic stagnation, the popularity of medical school has dramatically increased among pre-medical students in Japan. This is primarily due to the belief that medicine is generally a recession-proof career. As a result, pre-medical students today who want to enter medical school have to pass a more rigorous entrance examination than that in the 1980s. This paper explores the association between the selectivity of medical school admissions and graduates’ later career choices.

**Methods:**

A unique continuous measure of the selectivity of medical school admissions from 1980 to 2017, which is defined as the deviation value of medical schools, was merged with cross-sectional data of 122 990 physicians aged 35 to 55 years. The association between the deviation value of medical schools and various measures of physicians’ career choices was explored by logistic and ordinary least square regression models. Graduates from medical schools in which the deviation value was less than 55 were compared with those from more competitive medical schools, after controlling for fixed effects for the medical school attended by binary variables.

**Results:**

From 1980 to 2017, the average deviation value increased from 58.3 to 66.3, indicating a large increase in admission selectivity. Empirical results suggest that increasing selectivity of a medical school is associated with graduates having a higher probability of choosing a career in an acute hospital as well as having a lower probability of opening their own clinic and choosing a career in primary health care. Graduating from a highly competitive medical school (i.e., deviation value of more than 65) significantly increases the probability of working at typical acute hospitals such as so-called 7:1 hospitals (OR 1.665 2, 95%CI 1.444 0–1.920 4) and decreases the probability of working at primary care facilities (OR 0.602 6, 95%CI 0.441 2–0.823 0). It is also associated with graduates having a higher probability of becoming medical board certified (OR 1.294 6, 95%CI 1.108 8–1.511 4).

**Conclusion:**

Overall, this paper concludes that increased selectivity of medical school admissions predicts a higher quality of physicians in their own specialty, but at the same time, it is associated with a lower supply of physicians who go into primary care.

## Introduction

In many developed countries, the shortage of primary care physicians, who are not hospital residents and practice primary or first contact care in community settings [1], is commonly regarded as a serious policy issue [2, 3]. In response to this issue, some countries have implemented policy packages including the reform of medical school admissions criteria and curriculum [4]. Japan also implemented a major curriculum reform in 2004 and has adopted targeted admissions policies since 2009 with the goal of promoting the participation of young physicians in primary care and alleviating the geographic mal-distribution of primary care physicians [5, 6]. Also, a new board certification system for physicians’ clinical specialties was implemented in 2018, which officially acknowledged family medicine and general practice as a new discipline [7]. Despite these efforts, however, some point out that medical students’ attitudes toward primary care and chronically ill patients become more negative during medical school training [8–10]), and these attitudes influence students’ eventual choices of medical specialty [11–13].

With this in mind, some anecdotal evidence in Japan may indicate the reason that young physicians have a high interest in advanced medical techniques but are inattentive to family medicine [14, 15]. During Japan’s “lost decade” (i.e., the long-lasting recession beginning in 1991) [16], becoming a physician was regarded as a recession-proof career among high school students in Japan. As pointed out by Chen et al. [17], physicians’ careers are less subject to economic fluctuations than other high-paying occupations. Due to the nature of medicine as a safe and well-paying professional occupation, the total number of applicants to medical school in Japan is now far larger than the number of available admission slots. According to national statistics, the number of total applicants increased 2.5 times from 1980 to 2015, but the number of successful applications remained almost unchanged due to the strict controls set by the national government [18]. This has led to tremendous changes in the selectivity of medical school admissions. Over time, the intellectual standards required from applicants have dramatically increased, especially among medical schools that initially had low selectivity standards. For example, the deviation value of the Medical School of Juntendo University was just 50, but increased to 70 in 2017, which suggests that the test scores of students in this medical school increased by 2 standard deviations. Additionally, 65% of new students in private medical school utilized backdoor admissions to the school by offering monetary bribes during the 1970s [19], but now almost all new students enroll after rigorous screening.

While greater academic competence of medical students would improve the quality of health care in the future, it is not clear that those who obtain excellent test scores may have same career preferences compared with those who do not. Thus, this paper explores the broad consequences of such drastic changes in the characteristics of medical students by analyzing the census data of physicians as well as a unique index of medical school selectivity.

## Methods

### Physician database

Initially, we conducted a secondary analysis of the physician and dentist database complied by Nihon Ultmarc as of October 2017. Nihon Ultmarc gathers detailed information on clinically active physicians for medical representatives (MR), whose job is to meet with physicians and provide them information on pharmaceutical products. When MRs meet physicians, they report the physicians’ basic information to Nihon Ultmarc, who then immediately shares the information with all MRs registered in their system. In this way, Nihon Ultmarc successfully gathers information for almost all clinically active physicians in Japan. Recently, this database has been used in several medical science studies [20, 21]. The database lists each physician’s gender, birth year, graduation year, medical school attended, and year of establishing a clinic (if they did so). In addition, this database also provides detailed characteristics of the medical facilities where each physician practices. The total number of physicians and dentists in this database was 344 968 at the time of this study’s analysis.

### Institutional background

Throughout the entire admission process, one feature of medical school admission in Japan is the reliance on the cognitive-performance-based track. Some other developed countries use both a cognitive-performance-based track and an attribute-based track for medical school admission, but the share of medical students from the attribute-based track in Japan was less than 1% in 2005. It was 2010 when the proportion of new medical students from attribute-based track (i.e., regional quota track) exceeded 10% [22].

The actual process of the cognitive-performance-based track generally consists of three parts, namely, two paper tests and an interview. In the first step, many medical schools require students to take the National Center Test for University Admissions (NCTUA). If an applicant’s score on the NCTUA is below the required threshold, their application will be rejected. Otherwise, he/she can take a paper test, which differs by medical school. Finally, almost all medical schools require an interview in order to be evaluated on attributes that are not measured by paper tests. The final decision at each medical school is made by considering the results of both the second paper test and the interview.

In Japan, students enter medical school directly after graduation from high school. It is relatively rare for people who study other sciences in university to enter medical school afterwards. In our data, 84% of clinically active physicians entered medical school in the age range of 18 to 21 years. The timing of medical school entry is much earlier than that of students in the United States in America, where the average age of medical school applicants is 24 years [23]. After 6 years of medical school and 2 years of clinical training, medical students make their final career decisions. Grade retention and drop-outs are relatively rare in Japan; about 85% of medical students who entered medical school in 2013 graduated 6 years later [24].

### Index of admission selectivity

Note that it is very difficult to compare the selectivity of different medical schools because each one has a different paper test and a different interview process. However, some private tutoring schools administer mock entrance examinations and, from the results, create a general database that can be used as a proxy for the comparison of paper test selectivity. In this paper, an index of medical school admissions’ selectivity was obtained from the results of one such authoritative mock examination implemented by a large, nation-wide private tutoring school called Kawai-juku. The national mock examination organized by Kawai-juku, which started in 1972, is the largest mock examination in Japan, with 3.1 million students participating in 2017. By observing the results, Kawai-juku creates a continuous index of selectivity for the upcoming entrance examination. This index is calculated as a deviation value (in Japanese, *Hensachi*). The deviation value of individual *i* is given as below:

1 $$ {T}_i=\frac{10\left({x}_i-\mu \right)}{\sigma }+50, $$

where *σ* is standard deviation, *μ* is the mean, and *x*_*i*_ is the score of *i*. Note that this formula is close to that of the *z*-score, which is commonly used in quantitative analysis in many fields. The deviation value of a university is normalized to 50, which means the students who earn an average score on the national mock examination are estimated to have a 50% probability of success on the upcoming entrance examination. In a similar sense, the success probability of a student with a deviation value of 60 is 50% for a medical school with a deviation value of 60.

### Selectivity trends of medical school admissions since 1980

**Fig. 1:**
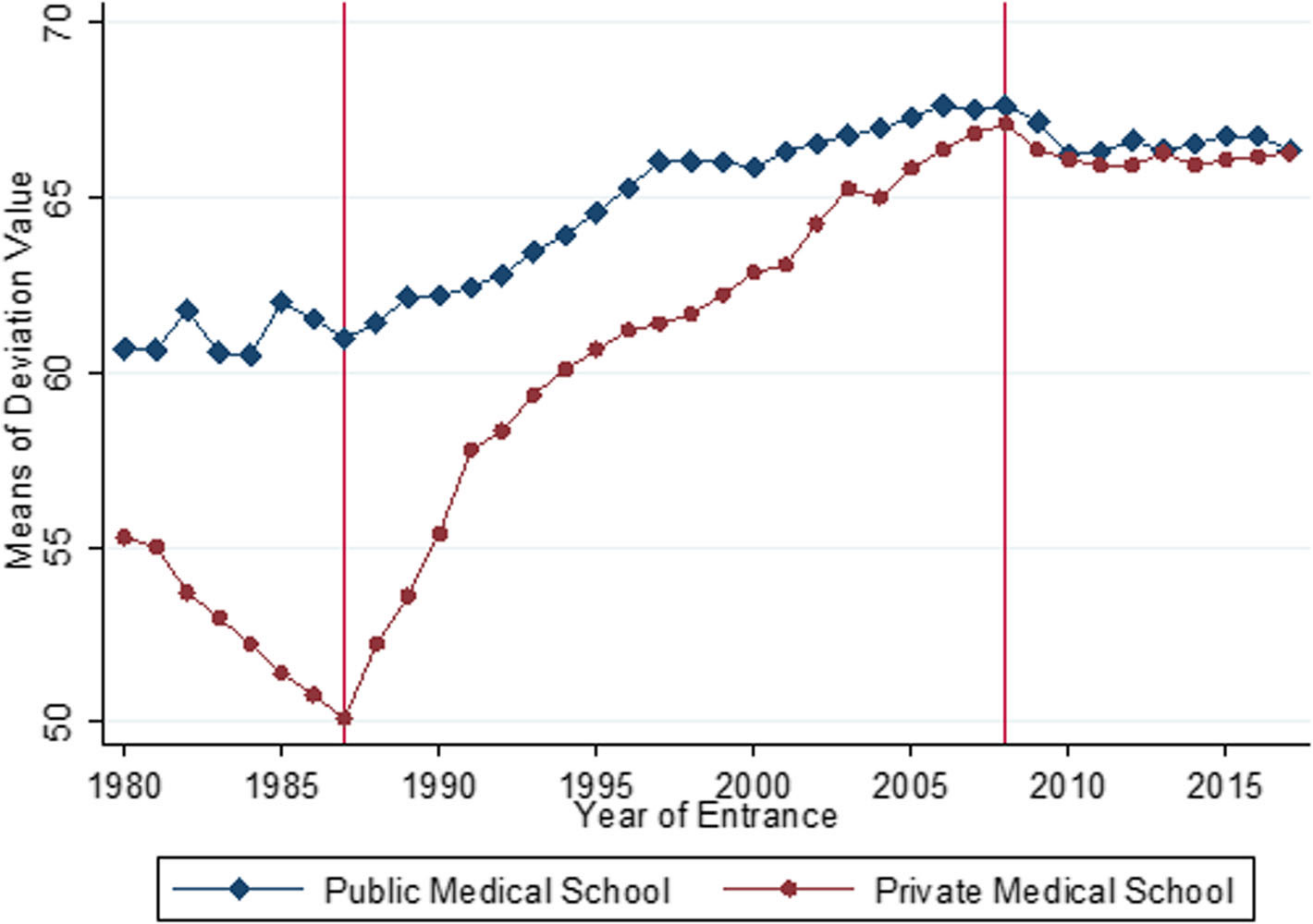
Deviation value of medical schools: public vs. private. Note: Deviation value (in Japanese, *Hensachi*) of each medical school, which is calculated by Kawai-juku, is a standard measure of the intellectual rank of a university’s entrance examination. Higher values indicate higher academic standards concerning the entrance examination. Vertical lines represent 1987 and 2009 when the major reforms of entrance examinations were implemented. Diamonds represent the mean of deviation values among public medical schools and circles represent the mean of deviation values among private medical schools. The number of private and public medical schools is 27 and 53, respectively. Finally, deviation values of each medical school from 1980 to 2015 are reported in Table 4 in the Appendix

Figure 1 plots the average deviation value of medical schools. Circles represent the mean of public medical schools, while diamonds represent the mean of private medical schools. The total number of medical schools included in this analysis is 79, including 27 private and 52 public medical schools. The number of medical schools in Japan is 80, and the deviation value of the National Defense Medical College is not calculated due to the uniqueness of its admissions process.

Overall, from 1980 to 2017, the average deviation value increased from 58.3 to 66.3, indicating a large increase in admission selectivity. Before 1987, the entrance examination in private medical schools was not so selective. In addition, application to medical school was a somewhat risky choice because applicants were prohibited from applying to multiple public medical schools at that time. Thus, if they were rejected by their public medical school of choice, they generally then had to apply to a private medical school that had much higher tuitions. However, the 1987 reform allowed for multiple applications to public medical schools, and as a result, the number of applications increased. Since applicants to public medical schools also continued applying to private medical schools, average deviation values in private medical schools also exhibited a sharp increase since 1987. While the 1987 reform played an important role in increasing the selectivity of admissions at both public and private medical schools, we should also pay close attention to the timing of the reform since it was very close to the bursting of the bubble economy in 1991. That year, the Japanese economy went into a long-lasting recession, the so-called lost decades [16]. Given that becoming a physician is regarded as a recession-proof career [17, 25], it is natural to think that the economic downturn contributed to the increasing competitiveness of medical school admissions [14]. In addition, even 10 years after the 1987 reform, deviation values exhibited a persistent upward trend. This suggests that a one-shot institutional change in 1987 cannot fully explain the overall upward trend in competitiveness from 1987 to 2008. In 2008, deviation values decreased slightly since the total number of new medical students, which is strictly regulated by the national government, was raised in order to address a nationwide shortage of physicians. A regional quota system for medical school admissions was also introduced at this time [21].

### Outcome variables

To explore the characteristics of physicians who graduated from medical schools with different levels of selectivity, three types of outcome variables were considered.

First, we evaluated choice of specialty and board certification. Medical specialty was classified using seven categories, namely, internal medicine, surgery, rehabilitation, neurology, radiology and anesthesiology, emergency and perinatal care, and other care. Emergency and perinatal care are grouped together because these specialties are generally unprofitable under the Japanese reimbursement system. Radiology and anesthesiology are also grouped together because the typical careers of radiologists and anesthesiologists are both hospital-oriented. In addition, we created a binary variable that takes a value of one if a physician is certified by the medical board of his or her specialty.

Second, we analyzed choice of practice location based on the municipality where each physician decided to practice as of 2017. In this analysis, basic municipality-level characteristics such as population density, income per capita, geriatric population (> 65 years old), and pediatric population (< 15 years old) were chosen as outcome variables. These outcomes are of particular importance because many countries consider the geographical mal-distribution of physicians a pressing issue, as physicians often do not choose rural areas to locate their practices [6, 26–28]. The selectivity of medical school admissions may be associated with this outcome.

Third, we assessed the impacts on medical-facility-level choices. In Japan, physicians start their careers in medical school hospitals and learn advanced medical techniques as residents. After experiencing many cases in medical school hospitals and other affiliated hospitals, they become independent by establishing their own clinics. Since the public reimbursement rate is set higher for primary care than hospital care [29], physicians generally have large monetary incentives to open their own clinics. Thus, a binary variable for the physicians who established their own clinics was created. In addition, we evaluated the likelihood of choosing a career in the “core” primary health care such as in home care support centers, long-term care facilities, and community health centers. In fact, the Japanese government has currently tried to increase the supply of home health care in order to prepare for a super aging society. For example, home care support clinics (in Japanese, *Zaitaku ryouyou shien shinryoujyo*) are those clinics with home care support functions available 24 h a day for terminally ill patients, regardless of specialty such as internal medicine and surgery [30].

In contrast, graduates from highly selective medical schools may exhibit strong preferences for a career in an acute hospital since they offer more opportunities to learn advanced medical techniques and to treat patients with more complex illnesses compared with long-term care hospitals. To evaluate the impacts of these preferences, two binary variables were created. The first was for physicians who work in hospitals with a 7:1 basic reimbursement rate (7:1 hospitals) and the second for those that adopt a diagnosis procedure combination/per-diem payment system (DPC hospitals). The most important criterion for the 7:1 basic reimbursement rate is to have more than 1 nurse for every 7 inpatients. In the Japanese reimbursement system, 7:1 hospitals are considered to be typical acute-care hospitals and are rewarded with higher reimbursement rates because they hire a larger number of registered nurses per patient and are expected to provide a higher intensity of care. In addition, DPC hospitals generally treat patients with acute conditions. Of note, many DPC hospitals are reimbursed at the 7:1 basic reimbursement rate. In 2014, the number of beds in 7:1 hospitals nationwide was about 380 000, which was less than that of DPC hospitals (480 000).

### Study population

The deviation values of medical schools from 1980 to 2017 were matched with physician-level data by using each physician’s medical school and the year of entrance into medical school. The entrance year was calculated by subtracting 6 years from the graduation year. Among matched samples, we excluded dentists for the purposes of this analysis. In addition, physicians aged 35 to 55 years were included and matched; physicians younger than 35 years old generally still belong to hospitals affiliated with their medical school, and physicians over 55 years old could not be matched with data on deviation values, and so these age groups were excluded. Eventually, after excluding missing values, 122 990 physicians were included in the empirical analysis.

### Statistical analysis

We employed a cross-sectional framework for analysis. While cross-sectional analysis is generally subject to several biases, it should be emphasized that the fixed effects of medical school attended are appropriately controlled for. Thus, our estimates capture the differential effects of entrance examination selectivity *within* medical schools. These differential effects seem to be plausibly identified because some medical schools that were initially high-ranked (e.g., the medical school of The University of Tokyo) did not experience a large increase in selectivity, but those that were initially low-ranked did. In addition, the inclusion of fixed effects for medical school attended improved the accuracy of our estimates. For example, applicants to one medical school today and those who applied a decade ago are thought to share some unobserved characteristics, such as geographical proximity to the medical school. These unobservable characteristics may affect later career choices. By controlling for fixed effects of medical school attended, the impact of potential bias from these unobserved factors can be diminished. Here, by using cross-sectional data of physicians as of October 2017, the following equation is estimated by logistic regression model:

2 $$ Ln\left[\frac{y_{imt}}{1-{y}_{imt}}\right]={\alpha}_0+{\alpha}_1{D}_1\left[55<{\mathrm{Rank}}_{mt}\le 60\right]+{\alpha}_2{D}_2\left[60<{\mathrm{Rank}}_{mt}\le 65\right]+{\alpha}_3{D}_3\left[{\mathrm{Rank}}_{mt}>65\right]+{\alpha}_4{X}_i+{\theta}_m+{\sigma}_i. $$

Here, *y*_*imt*_ is the outcome variable of physician *i* as of the survey year, who was enrolled in medical school *m* in year *t*. Rank_*mt*_ is the deviation value of medical school *m* in year *t*. *D*_1_[55 < Rank_*mt*_ ≤ 60] is a binary variable for physicians whose graduated medical school was located in 55 < Rank_*mt*_ ≤ 60. *D*_2_ and *D*_3_ are also understood in a similar manner. *α*_1_, *α*_2_, and *α*_3_ measures relative impacts of the competitiveness of entrance examination, when compared with *Rank*_*mt*_ ≤ 55. *X*_*i*_ is a vector of physicians’ characteristics, *θ*_*m*_ is the fixed effects of medical school attended, and *σ*_*i*_ is an error term. To address the hierarchical structure of the error term, standard errors are clustered at the level of medical school attended. As for the covariates, age and dummy variables for entrance year of medical school and gender are both controlled for. Statistical analysis was performed using STATA SP version 14.0.

When interpreting *α*_1_, *α*_2_, and *α*_3_, it is useful to note that the interpretation of the deviation value is especially intuitive when it takes multipliers of 5 or 10. For example, one standard deviation change in test score is translated into “10” changes in deviation value. Thus, *α*_2_, which is a coefficient of *D*_2_[60 < Rank_*mt*_ ≤ 65], may capture the characteristics of the students whose test score is higher than the mean by 1–1.5 standard deviations.

Because a logistic regression model is used for binary outcome variables, the results are reported in terms of odds ratio. However, the ordinary least square model is used for continuous outcome variables.

## Results

### Descriptive statistics

Descriptive statistics are presented in Table 1. In this table, the entire sample is split into graduates from initially low-ranked medical schools and those from initially high-ranked medical schools. Medical schools are classified into two groups according to their 1980 deviation value. If the 1980 deviation value was lower than the median value, graduates from these universities are categorized as “Graduated from low-ranked medical schools”. Otherwise, they are categorized as “Graduated from high-ranked medical schools”.

**Table 1:** Descriptive statistics

	Total		Graduated from low-ranked medical schools		Graduated from high-ranked medical schools		
	(1)	(2)	(3)	(4)	(5)	(6)	
	Mean	S.D.	Mean	S.D.	Mean	S.D.	
**Choice of clinical specialty**							
Internal medicine (a)	0.37	(0.48)	0.38	(0.49)	0.37	(0.48)	***
Surgery (a)	0.12	(0.32)	0.10	(0.30)	0.13	(0.33)	***
Rehabilitation (a)	0.09	(0.29)	0.09	(0.29)	0.09	(0.29)	***
Neurology (a)	0.03	(0.17)	0.03	(0.16)	0.03	(0.18)	***
Radiology and anesthesiology (a)	0.06	(0.24)	0.06	(0.24)	0.06	(0.25)	***
Emergency and perinatal care (a)	0.05	(0.22)	0.05	(0.22)	0.05	(0.22)	***
Other care (a)	0.27	(0.45)	0.29	(0.45)	0.27	(0.44)	***
Board certification (a)	0.81	(0.39)	0.77	(0.42)	0.84	(0.37)	***
**Choice of municipality**							
Population density (population per square kilometer)	4404.75	(5316.80)	4324.86	(5269.84)	4464.57	(5350.96)	***
Income per capita (million JPY per capita)	3.46	(1.01)	3.40	(0.94)	3.50	(1.06)	**
Share of geriatric population (> 65 years old)	0.25	(0.04)	0.26	(0.04)	0.25	(0.04)	***
Share of pediatric population (< 15 years old)	0.12	(0.01)	0.12	(0.01)	0.12	(0.01)	***
**Choice of medical facility**							
7:1 hospital (a)	0.41	(0.49)	0.36	(0.48)	0.45	(0.50)	***
DPC hospital (a)	0.58	(0.49)	0.52	(0.50)	0.64	(0.48)	***
Establishing own clinic (a)	0.22	(0.41)	0.27	(0.44)	0.18	(0.38)	***
Primary care facilities (a)	0.04	(0.19)	0.04	(0.19)	0.04	(0.19)	
**Basic characteristics**							
Age	45.75	(5.84)	45.76	(5.80)	45.75	(5.87)	***
Female (a)	0.23	(0.42)	0.28	(0.45)	0.19	(0.40)	***
Entrance year of medical school	1991.53	(6.15)	1991.69	(6.18)	1991.41	(6.13)	***
Graduated from private university (a)	0.35	(0.48)	0.56	(0.50)	0.19	(0.39)	***
Observation	122 990		52 662		70 328		

Note: (a) Binary variables. Medical schools are classified into two groups according to their 1980 deviation value. If the 1980 deviation value was lower than the median value, graduates from these universities are categorized as “graduated from low-ranked medical schools.” Otherwise, they are categorized as “graduated from high-ranked medical schools.” Results on the *t* test for mean differences between “low-ranked medical schools” and “high-ranked medical schools” are reported in the rightmost columns. Abbreviations: *7:1 hospital* hospital with a 7:1 basic reimbursement rate, *DPC hospital* hospital that adopts a diagnosis procedure combination/per-diem payment system, *S.D.* standard deviation. ***p < 0.01, **p < 0.05, and *p < 0.1, respectively

As for medical specialty among all physicians, 38% chose internal medicine as their primary specialty, and this share does not change by medical school rank. As for board certification, 77% of graduates from low-ranked medical schools obtained medical board certification for their respective specialty, while this share increased to 84% among graduates from high-ranked medical universities. For the characteristics of the municipality where physicians chose to practice, we did not find particular differences, but graduates from high-ranked medical schools were more likely to practice in an urban area. The largest differences found involved the choice of medical facilities. For example, 45% of physicians who graduated from high-ranked medical schools worked for 7:1 hospitals, while this share falls to only 36% for those from low-ranked medical schools. In addition, the latter group of physicians are more likely to establish their own clinics compared with the former group of physicians. Results of the *t* test for the mean comparison are reported in the rightmost column. Due to the large sample size, we find statistically significant differences in some variables even if there are no substantial differences. For example, income per capita and the population density in the municipality where each physician works is almost the same between the low and high groups.

### Regression results

Regression results on the choice of specialty and the probability to obtain board certification are reported in Table 2. Here, because the pattern of career choice may differ by gender [31], stratified results of male and female physicians are also presented. Board certification results are presented in column (1). The odds ratio of three binary variables of deviation value was consistently around 1.3 and statistically significant in the entire sample, suggesting that an increase of the deviation value enhances the probability of becoming medical board certified at the age from 35 to 55 years old. When compared with the graduates from medical schools in which the deviation value was less than 55, the odds ratio of the graduates from highly competitive medical schools in which the deviation values exceeded 65 was 1.294 6 (95%CI 1.108 8–1.511 4). In addition, we found similar increases in this outcome for both male and female physicians in panels A and B. Because our data surveyed whether each physician was certified by any of the 56 medical boards, we also investigated the effects on the certification from 7 major medical boards in columns (2) to (8). In panel A, we found significant positive association with deviation value only in radiology and anesthesiology (RA) and emergency and perinatal care (EP). In particular, the odds ratio of D1, D2, and D3 were large in the choice of radiology and anesthesiology. This probably reflected preference for high-tech care among graduates in highly competitive medical schools. However, we found no effects in internal medicine (column 2) and rehabilitation (column 4).

**Table 2:** Effects on board certification and choice of specialty

		Specialty						
	Board certification	IM	S	R	C	RA	EP	O
	(1)	(2)	(3)	(4)	(5)	(6)	(7)	(8)
**Panel A. Full sample**								
D1 [55 < Rank ≤ 60]	1.309 0***	1.01	0.917 1*	0.95	1.208 7**	1.227 0***	1.136 0**	0.953 6*
	[1.180 8–1.451 0]	[0.959 1–1.072 4]	[0.836 8–1.005 2]	[0.865 9–1.049 1]	[1.034 9–1.411 7]	[1.052 5–1.430 3]	[1.008 2–1.280 0]	[0.902 7–1.007 3]
D2 [60 < Rank ≤ 65]	1.307 0***	1.02	0.92	1.02	1.14	1.340 7***	1.187 5**	0.892 5***
	[1.152 8–1.481 8]	[0.944 7–1.100 5]	[0.819 3–1.026 0]	[0.914 3–1.145 1]	[0.907 5–1.423 3]	[1.114 4–1.612 9]	[1.001 2–1.408 4]	[0.825 4–0.965 2]
D3 [65 ≥ Rank]	1.294 6***	1.00	0.96	1.10	1.11	1.425 6***	1.247 5*	0.850 9**
	[1.108 8–1.511 4]	[0.880 1–1.128 4]	[0.833 5–1.104 1]	[0.938 4–1.286 5]	[0.819 1–1.498 2]	[1.138 1–1.785 7]	[0.981 2–1.586 2]	[0.740 0–0.978 6]
Obs.	122 693	122 252	122 252	122 252	122 252	122 252	122 252	122 252
Mean of dep.	0.81	0.37	0.12	0.09	0.03	0.06	0.05	0.28
**Panel B. Male physician**								
D1 [55 < Rank ≤ 60]	1.301 9***	1.060 9*	0.895 4**	0.942 8	1.202 9**	1.180 5	1.085 4	0.950 9
	[1.151 0–1.472 5]	[0.989 4–1.137 6]	[0.809 2–0.990 8]	[0.856 2–1.038 1]	[1.010 8–1.431 4]	[0.954 7–1.459 7]	[0.932 5–1.263 3]	[0.884 2–1.022 7]
D2 [60 < Rank ≤ 65]	1.302 8***	1.068 3	0.877 5**	1.029 4	1.093 1	1.306 9**	1.071 6	0.906 0**
	[1.126 6–1.506 7]	[0.969 5–1.177 3]	[0.776 9–0.991 1]	[0.917 4–1.155 0]	[0.858 5–1.392 0]	[1.025 3–1.665 8]	[0.872 1–1.316 7]	[0.825 3–0.994 7]
D3 [65 ≥ Rank]	1.312 8***	1.029	0.919 5	1.104 2	1.076 6	1.386 6**	1.19	0.863 1*
	[1.103 6–1.561 6]	[0.886 2–1.194 8]	[0.788 7–1.072 2]	[0.936 8–1.301 3]	[0.779 4–1.487 2]	[1.027 6–1.871 0]	[0.883 3–1.603 2]	[0.738 8–1.008 3]
Obs.	94 372	94 372	94 372	94 372	94 372	94 372	94 372	94 372
Mean of dep.	0.82	0.38	0.14	0.11	0.04	0.05	0.04	0.24
**Panel C. Female physician**								
D1 [55 < Rank ≤60]	1.287 2***	0.923 6	0.857 5	1.010 6	1.029 7	1.250 7**	1.256 7***	1.018 5
	[1.105 1–1.499 3]	[0.822 4–1.037 3]	[0.668 7–1.099 6]	[0.763 9–1.336 9]	[0.614 3–1.726 0]	[1.004 7–1.556 9]	[1.060 9–1.488 6]	[0.918 9–1.129 0]
D2 [60 < Rank ≤ 65]	1.260 3**	0.939	0.962 2	0.974 9	1.470 1	1.326 1*	1.402 3***	0.921 3
	[1.033 5–1.537 0]	[0.801 3–1.100 4]	[0.662 7–1.397 2]	[0.651 8–1.458 2]	[0.748 8–2.886 5]	[0.999 2–1.759 8]	[1.101 4–1.785 4]	[0.794 4–1.068 6]
D3 [65 ≥ Rank]	1.180 1	0.963 3	1.094 9	1.054 5	1.306 2	1.429 5*	1.322 2	0.861 6
	[0.908 8–1.532 4]	[0.758 4–1.223 5]	[0.623 8–1.921 8]	[0.593 7–1.872 8]	[0.478 1–3.568 6]	[0.994 3–2.055 3]	[0.918 1–1.904 2]	[0.699 6–1.061 1]
Obs.	27 880	27 880	27 880	27 880	27 880	27 880	27 880	27 880
Mean of dep.	0.78	0.36	0.05	0.03	0.01	0.09	0.08	0.38

Note: Results are based on logistic regression model. In all equations, the polynomial function of physician’s age, gender, and fixed effects of medical school attended are controlled for. Standard errors are clustered at the level of medical school attended. Abbreviations: *IM* internal medicine, *S* surgery, *R* rehabilitation, *N* neurology, *RA* radiology and anesthesiology, *EP* emergency and perinatal care, *O* other care. “Obs” represents the number of observations, “Mean of dep.” represents the mean of dependent variables. 95% confidence interval is in parentheses. ***p < 0.01, **p < 0.05, and *p < 0.1, respectively

Next, the results for municipality-level location choice and facility-level choice are summarized in Table 3. For municipality-level location choice, we found strong effects in column (1) among female physicians. Results in this column show that female physicians who graduated from highly competitive medical schools (D3) choose to practice in 17% more densely populated municipalities. This suggests high preference for city amenities among female physicians who graduated from highly competitive medical schools. However, no strong effects were found in the other dependent variables in columns (1) to (4) or among male physicians.

**Table 3:** Effects on practice location and medical facility choice

	Municipality-level choice				Facility-level choice			
	Ln population	Income	Elderly	Child	7:1	DPC	Clinic	Primary care
	Density		Share	Share			Owner	Facilities
	OLS	OLS	OLS	OLS	LOGIT	LOGIT	LOGIT	LOGIT
	(1)	(2)	(3)	(4)	(5)	(6)	(7)	(7)
**Panel A. Full sample**								
D1 [55 < Rank ≤ 60]	1.035 7	1.006 8	0.998 7*	1.000 1	1.519 9***	1.353 9***	0.840 9***	0.813 1**
	[0.975 7–1.099 4]	[0.966 4–1.048 9]	[0.997 2–1.000 1]	[0.999 4–1.000 7]	[1.388 4–1.663 9]	[1.232 2–1.487 6]	[0.780 1–0.906 4]	[0.681 4–0.970 3]
D2 [60 < Rank ≤ 65]	1.043 3	0.997 6	0.998 7	1.000 5	1.668 0***	1.470 9***	0.778 0***	0.724 6***
	[0.968 6–1.123 8]	[0.953 4–1.043 7]	[0.996 5–1.000 9]	[0.999 6–1.001 3]	[1.491 0–1.866 1]	[1.296 8–1.668 4]	[0.690 0–0.877 1]	[0.587 6–0.893 5]
D3 [65 ≥ Rank]	1.072 8	0.990 5	0.998 6	1.000 4	1.665 2***	1.473 2***	0.789 8***	0.602 6***
	[0.983 9–1.169 7]	[0.938 5–1.045 3]	[0.995 7–1.001 4]	[0.999 4–1.001 5]	[1.444 0–1.920 4]	[1.241 8–1.747 6]	[0.670 2–0.930 8]	[0.441 2–0.823 0]
Obs.	122 990	122 990	122 990	122 990	122 990	122 990	122 693	122 375
Mean of dep.	7.51	3.46	0.25	0.12	0.41	0.59	0.22	0.04
**Panel B. Male physician**								
D1 [55 < Rank ≤ 60]	0.028 7	0.000 6	− 0.001 5*	0.000 2	1.600 5***	1.411 6***	0.812 0***	0.869 4
	[− 0.040 4 to 0.097 7]	[− 0.044 4 to 0.045 6]	[− 0.003 2 to 0.000 2]	[− 0.000 4 to 0.000 9]	[1.444 8–1.772 9]	[1.267 4–1.572 1]	[0.752 0–0.876 7]	[0.709 2–1.065 7]
D2 [60 < Rank ≤ 65]	0.024 2	− 0.022 4	− 0.000 9	0.000 5	1.729 6***	1.529 1***	0.755 3***	0.819 3
	[− 0.056 3 to 0.104 7]	[− 0.070 2 to 0.025 4]	[− 0.003 3 to 0.001 5]	[− 0.000 4 to 0.001 5]	[1.537 9–1.945 2]	[1.328 1–1.760 5]	[0.668 0–0.853 9]	[0.625 1–1.073 8]
D3 [65 ≥ Rank]	0.041 7	− 0.038 5	− 0.001 1	0.000 6	1.715 6***	1.544 7***	0.752 7***	0.669 1*
	[− 0.051 0 to 0.134 4]	[− 0.096 5 to 0.019 6]	[− 0.004 1 to 0.001 8]	[− 0.000 6 to 0.001 8]	[1.481 1–1.987 3]	[1.274 3–1.872 3]	[0.635 1–0.892 0]	[0.443 6–1.009 2]
Obs.	94 849	94 849	94 849	94 849	94 849	94 849	94 849	94 849
Mean of dep.	7.44	3.41	0.26	0.12	0.42	0.60	0.24	0.04
**Panel C. Female physician**								
D1 [55 < Rank ≤ 60]	0.066 4	0.014 6	− 0.001 1	− 0.000 4	1.266 3***	1.156 8**	0.975	0.736 5**
	[− 0.016 5 to 0.149 2]	[− 0.043 7 to 0.072 9]	[− 0.003 1 to 0.001 0]	[− 0.001 5 to 0.000 8]	[1.123 2–1.427 7]	[1.020 3–1.311 6]	[0.825 3–1.151 8]	[0.570 1–0.951 5]
D2 [60 < Rank ≤ 65]	0.104 9**	0.051 7	− 0.002 6*	0.000 4	1.464 0***	1.278 8***	0.864	0.569 2***
	[0.000 6–0.209 2]	[− 0.025 7 to 0.129 1]	[− 0.005 5 to 0.000 3]	[− 0.000 9 to 0.001 8]	[1.246 8–1.719 0]	[1.101 2–1.4850]	[0.685 5–1.088 9]	[0.387 5–0.836 1]
D3 [65 ≥ Rank]	0.173 6**	0.080 6	− 0.002 7	0.000 2	1.484 3***	1.256 5**	0.965 3	0.477 0**
	[0.030 9–0.316 3]	[− 0.022 1 to 0.183 4]	[− 0.006 8 to 0.001 5]	[− 0.001 4 to 0.001 8]	[1.205 0–1.828 3]	[1.016 2–1.553 7]	[0.700 3–1.330 6]	[0.254 5–0.894 0]
Obs.	28 141	28 141	28 141	28 141	28 141	28 097	28 077	28 015
Mean of dep.	7.75	3.61	0.25	0.12	0.37	0.54	0.14	0.05

Note: In all equations, the polynomial function of physician’s age, gender, and fixed effects of medical school attended are controlled for. Standard errors are clustered at the level of medical school attended. Abbreviations: *7:1* hospital with a 7:1 basic reimbursement rate, *DPC* hospital that adopts a diagnosis procedure combination/per-diem payment system. “Obs” represents the number of observations, “Mean of dep.” represents the mean of dependent variables “OLS” represents ordinary least square and “LOGIT” is logistic regression Coefficient and odds ratio are reported in OLS and LOGIT model, respectively. 95% confidence interval is in parentheses. ***p < 0.01, **p < 0.05, and *p < 0.1, respectively

The results of medical facility choice are reported in columns (5) to (8). In this aspect, we found quite strong effects. For example, among male physicians, the odds ratio of D3 in the probability of working in a 7:1 hospital in column (5) was 1.665 2 (95%CI 1.444 0–1.920 4). In addition, because DPC hospitals also provide acute hospital care as in 7:1 hospitals, we also found large effects on the probability of working in a DPC hospital in column (6). In contrast, the probability of working in primary care sharply decreases according to the selectivity of the medical school of graduation. In column (7), the odds ratio of D3 for male physicians was 0.752 7 (95%CI 0.635 1–0.892 0), suggesting that the share of male physicians who establish their own clinics decreases greatly, when compared with graduates from non-competitive medical schools. This may be consistent with the findings of Mori [32] who showed physicians were less likely to have their own clinics if they were not the son of physicians and graduated from a public medical university. As a result of high persistence of having a career in acute hospitals and low intent to become primary care physicians, we find in column (8) that medical students, especially male medical students, who were successful in the entrance examination of a highly selective medical school, tend not to choose careers in primary care facilities such as home care clinics. In panel A, the odds ratio of D3 was 0.602 6 (95%CI 0.441 2–0.823 0). As to the differential effects by gender, stronger effects were found in male physicians than in female physicians. While statistical power was necessarily larger among male physicians due to the larger sample size, odds ratios among male physicians also seemed to be much more significant than among female physicians in columns (5) to (8).

Clearly, clinicians in some specialties (e.g., anesthesiologists) work at hospitals rather than in private clinics, so we implemented subsample analysis focused on internists. The results presented in Table 5 in the Appendix suggest that our main results still hold even in this subsample.

Finally, the same regression model is applied by age group (i.e., 35–45 years and 46–55 years) because the changes in deviation value in the long-term may be influenced by many factors, even when controlling for fixed effects of medical school attended. The results are presented in Table 6 in the Appendix. In short, the main results on medical facility choice in this study are sufficiently robust.

## Discussion

While super-aging societies such as Japan will need a much larger health care workforce for primary care and home health care in the near future, Japan’s experience during the last three decades suggests that increasing selectivity of medical school admissions is associated with graduates having a higher probability of working at acute hospitals rather than primary care facilities. One explanation of these results is that medical students who were most successful in the highly selective entrance examination have greater interest in advanced medical techniques and having a prestigious career in a large organization. This interpretation seems to be natural because previous studies have found that factors such as “rural background” [33], “lower job-related ambition” [34], and “lower interests in mastering advanced procedures” [7, 35] are all associated with medical students choosing to become family physicians or general practitioners. This point is noteworthy because, unlike other developed countries (e.g., Germany), there is no regional quota system of the number of primary care physicians. Given that several population estimates reveal shrinking populations in rural areas in the future, medical school graduates will be more likely to seek job opportunities in metropolitan areas. To alleviate shortage of primary care physicians in rural areas, it is particularly important for the Japanese government to take much stronger policy interventions on the entire physician career, as well as the system of medical school admissions.

One contribution of this paper is to provide evidence of an unintended consequence of cognitive-performance-based admission criteria (i.e., test score). Medical school admission in Japan has been heavily reliant on cognitive-performance-based criteria, but many countries have adopted multiple admission tracks for medical school in order to enroll young people who did not attain top grades during pre-university education, but have other valuable attributes [36], and organizing these alternative admission tracks is extremely policy-relevant. In fact, there is an extensive policy debate on the “best mix” of the cognitive-performance-based track and the attribute-based track for medical school admission. Accordingly, it is known that high entry scores are associated with performance after medical school admission and a lower probability of dropout [37, 38], but a disproportionately high reliance on the cognitive-performance-based track is also criticized because it narrows the diversity of medical students [36]. Given that, in Japan, there has been no tradition of open admission policy and university admission has been mainly determined by test scores, the Japanese experience in medical school admission is useful in understanding the pros and cons of the cognitive-performance-based track.

We should also pay close attention to the fact that the admissions process is only one determinant of a physicians’ entire career choice. For example, although this paper shows large impacts of the medical schools’ deviation value at the time of admission, which is an appropriate proxy of students’ cognitive performance, medical education after the admission is of particular relevancy on the later career choice [10]. Therefore, relative importance of multiple factors should be carefully re-examined in the actual process of policy making.

There are several limitations to our study. First, the study does not control for various time-varying characteristics of medical schools, such as curriculum changes at individual schools and students’ preferences (e.g., preferences on work-life balance). If these characteristics have correlations with the deviation value and outcome variables, our estimate may provide biased results, even after controlling for the fixed effects of medical school attended. However, the curriculum in Japanese medical schools seems to have been stabilized by the 2004 reform [39]. The cohort affected by this reform was in their late 30s in October 2017 (i.e., the date of our cross-sectional data). Thus, we checked the robustness of our main results by excluding them and found that the results remain unchanged.

Second, while the deviation value provided by Kawai-juku is one of the most popular indices on medical school selectivity in Japan, this value was calculated from the results of mock examinations. Thus, measurement error on the *true* selectivity, which may attenuate some estimated effects, is unavoidable. More importantly, medical students gradually chose their careers over their 6 years of medical education and their early career is strongly subject to the network within each medical school. In fact, many new graduates receive training in the hospitals of their medical school early in their careers, and therefore, each medical school can select applicants who will be good fits for their medical school hospitals during the admission process. If this is the case, the deviation value is also a proxy for medical schools’ “preference” for types of physicians educated, rather than students’ preference of later career choice.

Finally, our physician database includes only clinically active physicians. This sample selection does not cause serious bias on the empirical results because the decision to work as a clinician may be stable over time and does not fluctuate according to the macro-economic situation. However, given that some people with medical licenses devote their careers to academic research, the impacts of this kind of career choice should be evaluated in the future.

## Conclusion

Greater academic competence of medical students would improve the quality of health care in the future, but it is not clear that those who obtain excellent test scores may have same career preferences compared with those who do not. Using the census data of physicians merged with the unique index of the selectivity of medical school attended, this study explores how this index, namely deviation value, is associated with physicians’ career choice. Eventually, the increased selectivity of medical school admissions predicts a higher quality of physicians in their own specialty, but at the same time, it is associated with a lower supply of physicians who go into primary care.

## Data Availability

The datasets on the deviation value of medical schools are available from the corresponding author on reasonable request. Sharing of the physician data used in this study is prohibited due to the confidential nature of the dataset.

## Appendix

**Table 4:** Details of the deviation value of medical schools

Medical school ID	Prefecture	Ownership	1980	1985	1990	1995	2000	2005	2010	2015
1	Hokkaido	Public	62.5	65	65	67.5	67.5	70	67.5	67.5
2	Hokkaido	Public	60	62.5	60	62.5	65	67.5	65	65
3	Hokkaido	Public	57.5	57.5	60	65	65	67.5	62.5	67.5
4	Aomori	Public	60	60	60	60	65	67.5	67.5	67.5
5	Iwate	Private	57.5	52.5	52.5	57.5	62.5	65	65	65
6	Miyagi	Public	65	67.5	65	67.5	70	70	70	67.5
7	Akita	Public	60	57.5	57.5	60	65	65	62.5	62.5
8	Yamagata	Public	60	60	57.5	60	65	65	65	62.5
9	Fukushima	Public	60	60	57.5	62.5	65	67.5	62.5	65
10	Ibaraki	Public	60	60	62.5	62.5	62.5	65	65	67.5
11	Tochigi	Private	65	67.5	67.5	67.5	67.5	70	67.5	67.5
12	Tochigi	Private	50	50	55	60	60	62.5	62.5	62.5
13	Gunma	Public	60	60	62.5	62.5	65	67.5	65	65
14	Saitama	Private	47.5	45	50	57.5	60	62.5	65	62.5
15	Chiba	Public	62.5	65	65	62.5	67.5	67.5	70	70
16	Tokyo	Private	57.5	55	60	60	65	65	67.5	65
17	Tokyo	Private	65	57.5	60	62.5	65	67.5	70	67.5
18	Tokyo	Public	70	70	70	70	70	72.5	72.5	72.5
19	Tokyo	Private	55	50	57.5	62.5	65	70	70	70
20	Tokyo	Public	65	67.5	67.5	67.5	70	70	70	70
21	Tokyo	Private	70	70	70	70	70	72.5	72.5	72.5
22	Tokyo	Private	60	55	60	65	67.5	70	67.5	67.5
23	Tokyo	Private	52.5	52.5	55	57.5	62.5	62.5	65	65
24	Tokyo	Private	60	60	60	62.5	65	70	70	70
25	Tokyo	Private	60	50	55	62.5	62.5	65	67.5	67.5
26	Tokyo	Private	57.5	52.5	57.5	57.5	62.5	65	67.5	67.5
27	Tokyo	Private	47.5	42.5	47.5	60	60	65	67.5	65
28	Tokyo	Private	50	45	52.5	60	60	65	65	62.5
29	Tokyo	Private	47.5	47.5	52.5	57.5	62.5	65	65	65
30	Kanagawa	Public	60	62.5	62.5	67.5	67.5	67.5	67.5	67.5
31	Kanagawa	Private	52.5	47.5	47.5	57.5	62.5	65	62.5	62.5
32	Kanagawa	Private	52.5	45	55	60	65	67.5	65	65
33	Niigata	Public	60	62.5	62.5	62.5	65	67.5	65	65
34	Toyama	Public	57.5	60	62.5	62.5	62.5	65	62.5	65
35	Ishikawa	Public	62.5	62.5	65	65	67.5	67.5	67.5	65
36	Ishikawa	Private	47.5	42.5	45	55	62.5	62.5	65	65
37	Fukui	Public	60	60	60	62.5	62.5	65	62.5	65
38	Yamanashi	Public	57.5	60	62.5	65	67.5	70	70	67.5
39	Nagano	Public	60	62.5	62.5	70	70	67.5	62.5	65
40	Gifu	Public	60	60	60	65	65	67.5	67.5	67.5
41	Shizuoka	Public	60	62.5	62.5	62.5	65	67.5	65	65
42	Aichi	Public	65	67.5	67.5	67.5	70	70	70	67.5
43	Aichi	Public	60	62.5	62.5	67.5	67.5	67.5	65	67.5
44	Aichi	Private	47.5	37.5	50	60	60	65	65	65
45	Aichi	Private	50	40	47.5	60	57.5	65	65	65
46	Mie	Public	60	60	62.5	65	65	65	65	67.5
47	Shiga	Public	57.5	60	62.5	65	62.5	65	65	67.5
48	Kyoto	Public	67.5	70	70	70	70	72.5	72.5	72.5
49	Kyoto	Public	67.5	65	65	65	67.5	70	65	70
50	Osaka	Private	57.5	55	57.5	62.5	62.5	65	65	70
51	Osaka	Private	60	60	60	65	65	67.5	67.5	70
52	Osaka	Public	67.5	67.5	67.5	70	70	72.5	70	72.5
53	Osaka	Public	62.5	65	62.5	65	67.5	70	65	67.5
54	Osaka	Public	57.5	57.5	57.5	60	62.5	65	67.5	67.5
55	Hyogo	Public	65	65	65	67.5	67.5	65	67.5	67.5
56	Hyogo	Private	55	52.5	55	60	62.5	65	62.5	65
57	Nara	Public	60	62.5	62.5	65	65	67.5	65	67.5
58	Wakayama	Public	60	62.5	60	65	65	67.5	65	67.5
59	Tottori	Public	57.5	60	60	62.5	65	65	65	65
60	Shimane	Public	57.5	57.5	60	65	65	65	65	65
61	Okayama	Public	62.5	65	65	67.5	67.5	67.5	65	67.5
62	Okayama	Private	47.5	47.5	50	55	57.5	62.5	62.5	62.5
63	Hiroshima	Public	62.5	62.5	62.5	67.5	65	67.5	67.5	67.5
64	Yamaguchi	Public	60	62.5	60	62.5	65	67.5	65	67.5
65	Tokushima	Public	57.5	62.5	60	67.5	65	67.5	65	65
66	Kagawa	Public	60	57.5	60	60	62.5	65	65	65
67	Ehime	Public	57.5	60	60	60	65	67.5	65	65
68	Kochi	Public	57.5	60	60	65	62.5	65	66.3	65
69	Fukuoka	Private	57.5	50	55	60	62.5	65	65	65
70	Fukuoka	Public	67.5	67.5	67.5	67.5	70	70	70	67.5
71	Fukuoka	Public	55	50	52.5	60	62.5	65	65	65
72	Fukuoka	Private	62.5	57.5	60	62.5	62.5	65	65	67.5
73	Saga	Public	57.5	60	62.5	n.	n.	65	n.	65
74	Nagasaki	Public	60	62.5	60	65	65	67.5	67.5	67.5
75	Kumamoto	Public	60	62.5	65	65	67.5	67.5	67.5	67.5
76	Oita	Public	57.5	60	60	62.5	67.5	65	65	65
77	Miyazaki	Public	57.5	60	60	62.5	62.5	65	65	65
78	Kagoshima	Public	57.5	62.5	62.5	65	62.5	65	65	65
79	Okinawa	Public	n.	60	60	60	65	65	65	65

Note: The National Defense Medical College is excluded because the deviation value is not calculated due to its unique admission process

**Table 5:** Effects on medical facility choice: internal medicine vs other specialty

	7:1	DPC	Clinic	“Core”
			Owner	Primary care
	LOGIT	LOGIT	LOGIT	LOGIT
	(5)	(6)	(7)	(7)
**Panel A. Internal medicine**				
D1 [55 < Rank ≤ 60]	1.511 8***	1.383 9***	0.860 1***	0.906 2*
	[1.327 4–1.721 9]	[1.226 3–1.561 7]	[0.775 3–0.954 2]	[0.807 4–1.016 9]
D2 [60 < Rank ≤ 65]	1.671 3***	1.492 0***	0.793 3***	0.889 3
	[1.415 1–1.973 9]	[1.275 9–1.744 7]	[0.686 7–0.916 4]	[0.740 8–1.067 5]
D3 [65 ≥ Rank]	1.689 0***	1.539 1***	0.795 4**	0.935 7
	[1.370 2–2.081 8]	[1.251 1–1.893 5]	[0.650 5–0.972 5]	[0.743 3–1.178 0]
Obs.	46 036	46 036	45 905	45 947
Mean of dep.	0.40	0.56	0.22	0.12
**Panel B. Other specialty**				
D1 [55 < Rank ≤ 60]	1.515 2***	1.336 7***	0.832 8***	1.076 3
	[1.383 9–1.659 0]	[1.207 5–1.479 7]	[0.759 8–0.912 8]	[0.956 2–1.211 5]
D2 [60 < Rank ≤ 65]	1.663 3***	1.461 4***	0.768 7***	1.090 1
	[1.488 1–1.859 3]	[1.267 1–1.685 4]	[0.664 3–0.889 5]	[0.922 1–1.288 7]
D3 [65 ≥ Rank]	1.654 0***	1.435 5***	0.783 6**	1.336 1**
	[1.416 9–1.930 6]	[1.166 5–1.766 4]	[0.638 3–0.962 1]	[1.008 1–1.770 8]
Obs.	76 954	76 954	76 652	76 614
Mean of dep.	0.42	0.60	0.22	0.04

Note: In all equations, the polynomial function of physician’s age, gender, and fixed effects of medical school attended are controlled for. Standard errors are clustered at the level of medical school attended. Abbreviations: *7:1* hospital with a 7:1 basic reimbursement rate, *DPC* hospital that adopts a diagnosis procedure combination/per-diem payment system. “Obs” represents the number of observations. “Mean of dep.” represents the mean of dependent variables. “LOGIT” represents logistic regression. Odds ratio are reported. 95% confidence interval is in parentheses. ****p* < 0.01, ***p* < 0.05, and **p* < 0.1, respectively

**Table 6:** Effects on medical facility choice by age groups

	7:1	DPC	Clinic	"Core"
			Owner	Primary Care
	LOGIT	LOGIT	LOGIT	LOGIT
	(5)	(6)	(7)	(7)
**Panel A. Age 35–45**				
D1 [55 < Rank ≤ 60]	1.165 1**	1.135 3*	0.811 2**	1.220 8
	[1.030 0–1.317 8]	[0.982 0–1.312 6]	[0.683 4–0.963 0]	[0.957 6–1.652 0]
D2 [60 < Rank ≤ 65]	1.366 9***	1.386 9***	0.690 4***	1.314 6
	[1.190 9–1.568 9]	[1.177 8–1.633 2]	[0.566 2–0.841 8]	[0.939 0–1.865 9]
D3 [65 ≥ Rank]	1.419 9***	1.406 3***	0.637 9***	1.424 7
	[1.205 5–1.672 3]	[1.159 7–1.705 3]	[0.489 3–0.831 6]	[0.928 8–2.132 3]
Obs.	52 078	52 077	51 594	51 885
Mean of dep.	0.49	0.73	0.10	0.05
**Panel B. Age 46–55**				
D1 [55 < Rank ≤ 60]	1.371 1***	1.360 4***	0.767 5***	0.840 0**
	[1.198 1–1.568 9]	[1.236 5–1.496 8]	[0.685 0–0.859 9]	[0.726 8–0.970 8]
D2 [60 < Rank ≤ 65]	1.483 6***	1.434 0***	0.721 4***	0.767 7**
	[1.233 0–1.785 1]	[1.232 5–1.668 5]	[0.601 5–0.865 2]	[0.620 2–0.950 3]
D3 [65 ≥ Rank]	1.470 6***	1.516 0***	0.647 2***	0.715 7
	[1.177 5–1.836 6]	[1.2203–1.883 5]	[0.503 8–0.831 3]	[0.474 6–1.079 3]
Obs.	32 138	32 138	32 114	32 067
Mean of dep.	0.39	0.53	0.25	0.08

Note: In all equations, the polynomial function of physician’s age, gender, and fixed effects of medical school attended are controlled for. Standard errors are clustered at the level of medical school attended. Abbreviations: *7:1* hospital with a 7:1 basic reimbursement rate, *DPC* hospital that adopts a diagnosis procedure combination/per-diem payment system. “Obs” represents the number of observations, “Mean of dep.” represents the mean of dependent variables. “LOGIT” represents logistic regression. Odds ratio are reported. 95% confidence interval is in parentheses. ****p* < 0.01, ***p* < 0.05, and **p* < 0.1, respectively

## Abbreviations

7:1 hospital: Hospital with a 7:1 basic reimbursement rate
DPC hospital: Hospital that adopts a diagnosis procedure combination/per-diem payment system
S.D: Standard deviation
IM: Internal medicine
S: Surgery
R: Rehabilitation
N: Neurology
RA: Radiology and anesthesiology
EP: Emergency and perinatal care
O: Other care
Dep.: Dependent variable
Obs.: Observations
OLS: Ordinary least square
LOGIT: Logistic regression
